# Evaluation of *Glycine max *mRNA clusters

**DOI:** 10.1186/1471-2105-6-S2-S7

**Published:** 2005-07-15

**Authors:** Ronald L Frank, Fikret Ercal

**Affiliations:** 1 Biological Sciences Department, University of Missouri-Rolla, Rolla, MO, USA; 2 Computer Science Department, University of Missouri-Rolla, Rolla, MO, USA

## Abstract

**Background:**

Clustering the ESTs from a large dataset representing a single species is a convenient starting point for a number of investigations into gene discovery, genome evolution, expression patterns, and alternatively spliced transcripts. Several methods have been developed to accomplish this, the most widely available being UniGene, a public domain collection of gene-oriented clusters for over 45 different species created and maintained by NCBI. The goal is for each cluster to represent a unique gene, but currently it is not known how closely the overall results represent that reality. UniGene's build procedure begins with initial mRNA clusters before joining ESTs. UniGene's results for soybean indicate a significant amount of redundancy among some sequences reported to be unique mRNAs. To establish a valid non-redundant known gene set for *Glycine max *we applied our algorithm to the clustering of only mRNA sequences. The mRNA dataset was run through the algorithm using two different matching stringencies. The resulting cluster compositions were compared to each other and to UniGene. Clusters exhibiting differences among the three methods were analyzed by 1) nucleotide and amino acid alignment and 2) submitting authors conclusions to determine whether members of a single cluster represented the same gene or not.

**Results:**

Of the 12 clusters that were examined closely most contained examples of sequences that did not belong in the same cluster. However, neither the two stringencies of PECT nor UniGene had a significantly greater record of accuracy in placing paralogs into separate clusters.

**Conclusion:**

Our results reveal that, although each method produces some errors, using multiple stringencies for matching or a sequential hierarchical method of increasing stringencies can provide more reliable results and therefore allow greater confidence in the vast majority of clusters that contain only ESTs and no mRNA sequences.

## Background

ESTs are generated when large numbers of randomly selected cDNA clones from various tissues, genotypes, developmental stages, or treatments are partially sequenced. The greater the number of ESTs generated from independently constructed libraries the more information can be derived from *in silico *analyses. These short single-pass sequences can be accumulated rapidly from the high-throughput sequencing methods developed for genome sequencing. Therefore, generating ESTs from many different tissues, genotypes, and conditions should increase the probability of at least one EST from each gene of an organism's genome. Subsequent grouping of those ESTs into gene-oriented clusters should theoretically produce one cluster for each unique gene.

A significantly large EST dataset can provide qualitative information regarding mRNA processing as well. Alternate forms of mature mRNA transcripts can originate from the same gene by a variety of known mechanisms. Alternate promoters, alternate polyadenylation sites [1,2], and alternative splicing of exons [3] can produce mRNAs that encode different but related protein functions. ESTs representing such transcripts can be aligned and compared to identify putative transcriptional and processing alternatives.

We have previously reported on the development of a fast and efficient algorithm, PECT (Parallel EST Clustering Tool), to cluster ESTs based on sequence matching [4]. An increasingly common use of EST datasets is the identification of multigene families [5]. An inherent difficulty in understanding the origin of gene families and their role in genome evolution is distinguishing between paralogs (family members originating by duplication events), homologs (alleles), and in the case of plants, homeologs (formerly orthologs separated by speciation events restored to a common genome by hybridization events). We are currently developing an algorithm to cluster ESTs in a hierarchical manner using matching criteria of increasing stringencies. By keeping track of each EST's membership from larger clusters to increasingly smaller clusters we hope to provide information on gene family relationships.

Clustering protocols must take into consideration the limitations inherent in the generation of ESTs. Since ESTs originate from single sequence experiments (single-pass sequences) errors that are normally eliminated by repeated sequencing of both strands of a given length of DNA remain in the EST. The nature of the sequencing method also results in a higher frequency of errors at the far end of the read. Truncating the sequence to eliminate these regions can result in higher average quality but shorter sequences. Highly expressed genes will be represented by many ESTs and often sequence ambiguities can be resolved by overlap. Analysis of the validity of the clusters produced has become an area of study in itself.

The accuracy of clustering methods is very hard to measure because the majority of the putative genes represented by clusters are as yet undiscovered or at least uncharacterized. UniGene, the most widely available clustered set uses a build procedure that begins by clustering mRNA sequences from the gene databank. The ESTs are then joined to existing mRNA clusters. New clusters are created for ESTs that do not join an mRNA cluster. This procedure presents two problems. One is the accuracy of the initial mRNA clusters. Do the mRNA sequences now found in the same cluster really originate from the same gene? If the original clusters are representative of more than one gene then the ESTs that join those clusters will result in significant overclustering, joining sequences that do not belong in the same cluster. The second problem is that two separate confidence levels exist for the clusters. Those clusters with no mRNA members may not be as reliable at representing existing genes as those with mRNA members. However, our approach is to cluster ESTs alone without mRNA sequences. The resulting clusters can then be compared to existing mRNA sequences to assess the accuracy with which the process assembled sequences from a known gene. This provides some measure of confidence in the majority of clusters for which no mRNA has yet been isolated. The first step in this process, however, requires a valid non-redundant set of known genes from the available mRNA sequences in the database. The fact that UniGene generated less than 700 clusters from about 850 soybean mRNA sequences indicates that either there exists a significant amount of redundancy in the dataset or many genes are being clustered together incorrectly.

To provide an indication of the performance of our algorithm, and to begin the process of generating a valid non-redundant set of genes for soybean, we have used PECT to cluster known soybean sequences (mRNAs), compared the results to UniGene, and analyzed the results by alignment of clustered sequences as well as assessing the authors' conclusions in the references cited for each submission, if available. Soybean provides a good beginning dataset for experimentation because as a crop species it is highly inbred and the number of ESTs is very large but not too large (350,000 versus 6 million for human or 4 million for mouse) for multiple runs of an algorithm such as PECT at varying stringencies. Allelic differences should be minimized because of the relatively few genotypes represented among the cDNA libraries used to generate ESTs (100 libraries of 20 genotypes versus 8000 libraries for human, virtually all from different genotypes). For the human genome noise due to allelic differences could be very high and make it very difficult to distinguish between some allelic variants and paralogs. For soybean homozygosity is high and noise due to allelic differences should be at a minimum. Once completed for an inbred organism like soybean and automation of the analysis steps, generating a valid non-redundant gene set from mRNA sequences by this method can enhance verification of EST clusters for any species represented in the NCBI EST database (dbEST).

## Results and Discussion

### Profile of Cluster Results

Table 1 shows the cluster profile of PECT at WS100 and WS200 (See Methods). As expected the total number of clusters increases (632 to 688) with increasing stringency of the match criterion. At the same time, the number of non-singleton (NS) clusters decreased from 113 to 86 indicating a net release of singletons from larger clusters. This is also an expected result of the application of a higher stringency.

**Figure 1:**
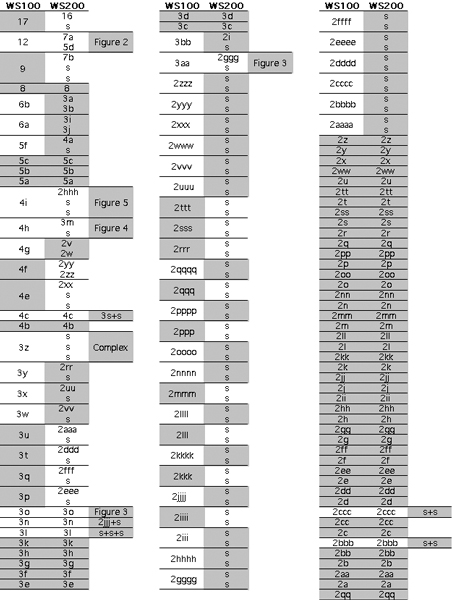
**Relative Distribution of All Non-Singleton Clusters. **Clusters are arranged in descending size according to WS100. Corresponding WS200 clusters are shown in the second column. Gray shaded clusters indicate UniGene agreement. Clusters designations correspond to cluster size with letters distinquishing between clusters of the same size, e.g., 6a and 6b refer to separate clusters of size 6. An *s *indicates a singleton (cluster of size one).

The left column of Figure 1 lists all the non-singleton (NS) clusters using at WS100 and arranged in descending cluster size. The second column shows the result of applying PECT at WS200 to the same dataset. Of the 113 NS clusters created at WS100, 61 remained unchanged, 44 had one sequence removed as a singleton (32 of which were originally cluster size 2), 3 clusters had two sequences removed as singletons (Clusters 9, 4e, and 4i), and 5 clusters were split into two smaller NS clusters (Clusters 12, 6a, 6b, 4f and 4g).

### Comparison to UniGene

**Figure 2:**
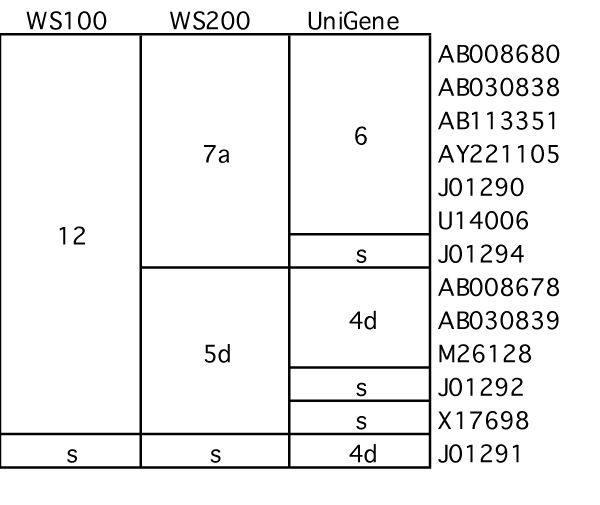
**Cluster 12. **Distribution of the 13 mRNA sequences of which 12 are WS100 Cluster 12. See Figure 1 legend for explanation of cluster designations. The right column corresponds to sequence accession numbers.

**Figure 3:**
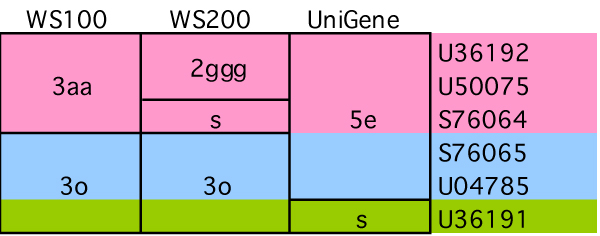
**Cluster 5e. **Distribution of the six mRNA sequences of which five are UniGene Cluster 5e. Shaded colors represent mRNA sequences from the same gene as determined by alignment analysis and literature review.

**Figure 4:**
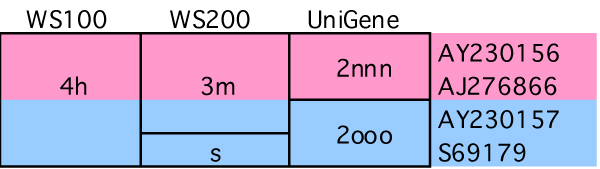
**Cluster 4h. **Distribution of the four mRNA sequences of WS100 Cluster 4h. Shaded colors represent mRNA sequences from the same gene as determined by alignment analysis and literature review.

**Figure 5:**
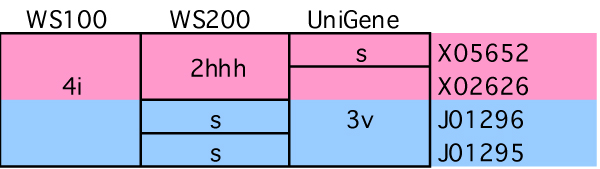
**Cluster 4i. **Distribution of the four mRNA sequences of WS100 Cluster 4i. Shaded colors represent mRNA sequences from the same gene as determined by alignment analysis and literature review.

The gray-shaded clusters in Figure 1 indicate where UniGene clusters match those generated by PECT. The 9 UniGene clusters that match neither the WS100 or WS200 stringencies and exhibit a more complex distribution of sequences among clusters are shown in Figures 2, 3, 4 and 5. Of particular interest is the fact that 83 of UniGene's 92 NS clusters (Table 1) agree with one or the other of the two stringencies applied by PECT. This broad agreement indicates a sequence matching algorithm can quickly generate a similar grouping of genes. A much closer analysis of the largest clusters (size 4 or greater), detailed below was necessary to reveal the relative accuracy of the clustering methods.

### Cluster 12

Cluster 12 contains mRNA sequences encoding conglycinin storage protein subunits. Sequence similarities are shared between the three subunit classes, α, α', and β, that themselves are composed of multigene families. Therefore, the type of sequence comparison performed here does not provide additional information as to the likely distribution of mRNA sequences to specific subunits. However, it is clear from a review of the literature cited for these sequences [6-9] that this single cluster contains representatives of both the α and α' subunit multigene families.

Cluster 12 of WS100 probably contains either two separate genes or perhaps even two separate multigene families. The conglycinin seed storage protein subunits exhibit enough sequence similarity to make absolute differentiation at this level of analysis difficult. WS200 put 6 of 7 sequences identified as α' by the authors into the same cluster and 5 of 7 identified as α into one cluster plus a singleton and therefore appeared to separate the two gene classes more closely than either WS100 or UniGene. See Figure 2.

### Cluster 9

Cluster 9 contains mRNA sequences encoding uricase. Sequence comparisons indicate that six (AB002810, D86929, D86930, M63743, M87019, M95400) could represent the same gene. Differences were very few and could be sequence errors. The literature cited for these sequences [10-12] support the identity of four of the six. The other two are unpublished direct submissions.

AB002809 likely represents a unique uricase gene. Among the 30 differences to D86929 in the coding region 7 are 1st position, 2 are 2nd position, and 21 are 3rd position (p < 0.005). Position difference analysis also suggests that L00353 represents a unique uricase gene. Comparison to D86929 reveals six differences in the coding region, 1 is 1st position, 0 are 2nd position, and 5 are 3rd position (p < 0.05). This is consistent with the reference citation [10]. X54365 is distinct from the others because it originates from the antisense strand, also consistent with the original report [12].

Cluster 9, the same for WS100 and UniGene, probably represents three distinct uricase genes and a sequence that is encoded from the antisense strand of a uricase gene. WS200 was closest to being correct because it clustered the six mRNA sequences that represent one gene while keeping as singletons a second gene and antisense RNA. However, WS200 did include the third uricase gene as part of the first genes cluster. See Figure 1.

### Cluster 6a

Cluster 6a contains mRNA sequences encoding ascorbate peroxidase. Pairwise sequence comparisons suggest two distinct genes represented by three mRNA sequences each, AB082930, AB082931, L10292 and AB082932, AF127804, U56634. Among the first group, AB082930 and AB082931 exhibit 1 base difference and 1 base gap in the coding region, but are identical in both the 5' and 3' UTR, while AB082931 and L10292 are identical. Similarly among the second group, AF127804 and AB082932 exhibit 99% identity where 1 difference is 1st position, 1 is 2nd, 4 are 3rd (p > 0.05, 0.299) and AB082932, U56634 are 99% identical with the only difference being a GC in one is CG in the other. Both 5' and 3' UTR sequences are identical. Conversely, upon comparison between the two groups, AF127804 and L10292 for example, the differences distribute as 7 in the 1st position, 6 in the 2nd, and 28 in the 3rd (p < 0.005). The literature cited for these sequences [13,14] confirm the above analysis except that the annotation for AF127804 denotes the gene name for the other group.

The six sequences in the WS100 Cluster 6a likely represent two separate ascorbate peroxidase genes. Both WS200 and UniGene had the sequences separated consistent with this conclusion. See Figure 1.

### Cluster 6b

Cluster 6b contains mRNA sequences encoding glycinin storage protein subunits. As is the case with conglycinin above, sequence similarities are shared between the subunit classes. However, pairwise comparisons did separate the mRNA sequences into two groups of three, AB113350, D00216, X02806, and AB113349, M36686, X02985. Within the first group, AB113350 and X02806 exhibit 1 difference in the 1st position, 1 in the 2nd, and 3 in the 3rd (p > 0.05, 0.473), while AB113350 and D00216 have only 1 difference in the 1639 base coding region and identical 3'UTR. Similarly within the second group, X02985 and AB113349 show differences distributed as 1 in the 1st position, 3 in the 2nd, and 3 in the 3rd (p > 0.05, 0.585), none in 3' UTR, while M36686 and AB113349 are identical. Conversely, between group comparison, AB113350 and AB113349 for example, exhibits 3 gaps in the coding region that maintain the reading frame. A review of the literature cited for these sequences [15-18] also indicates that these mRNA sequences represent two separate classes of glycinin subunit genes.

The six sequences in the WS100 Cluster 6b probably represent two separate glycinin seed storage protein subunit genes or two separate multigene families. Both WS200 and UniGene had the sequences separated consistent with this conclusion. See Figure 1.

### Cluster 5e

Cluster 5e contains mRNA sequences encoding lipoxygenase. Sequence comparisons suggest three distinct genes, the first represented by three, S76064, U36192, U50075, the second by two, S76065, U04785, and the third by one mRNA, U36191. Among the first group, S76064 and U36192 only overlap by 113 bases of the 340 and exhibit 4 base differences, 3 of which are TTT vs AAA, while U50075 and U36192 are 100% identical over 43 amino acids of the coding region and 200 bases of the 3' UTR. The second group, S76065 and U04785, are 100% identical. The third gene, U36191 when compared to a member of the first group, S76064, exhibits differences distributed as 11 in the 1st position, 4 in the 2nd, and 17 in the 3rd (p < 0.05). Likewise, when U36191 is aligned with a member of the second group, U04785, the differences distribute as 44 in the 1st position, 15 in the 2nd, and 70 in the 3rd (p < 0.005). Lastly, the comparison between members of group one and two, S76064 and S76065, respectively, the differences are distributed as 3 in the 1st, 1 in the 2nd, and 10 in the 3rd (p < 0.01). The literature cited for these sequences [19-21] also indicates a similar grouping of distinct lipoxygenase genes.

The five mRNA sequences in Cluster 5e of UniGene likely represent two distinct lipoxygenase genes. WS100 correctly separated the two groups but incorrectly included a third lipoxygenase gene in one of the clusters. UniGene had correctly kept that gene sequence separate as a singleton. WS200 was less accurate in keeping as a singleton an mRNA that should cluster with two others. See Figure 3.

### Cluster 5f

Cluster 5f contains mRNA sequences encoding a ribulose-1, 5-bisphosphate carboxylase small subunit. Pairwise comparisons among the five, AF303939, AF303940, AF303941, U39567, X54216, indicate that all represent the same gene. The literature is limited [22] as three of the sequences are unpublished direct submissions, but one paper suggests that two of the sequences represent homologous members of the SSU gene family.

All members of WS100 Cluster 5f represent small subunit genes of ribulose-1, 5-bisphosphate carboxylase, but whether or not they originate from the same gene or very similar paralogs is not clear. The authors [22] note that similarity in expression patterns illustrates coordinate evolution and supports the suggestion that gene conversion may homogenize the coding regions of these genes. The SSU genes may represent a good example of the need for clustering at multiple stringencies. See Figure 1.

### Cluster 4c

Cluster 4c contains mRNA sequences encoding the ferritin iron storage protein. Sequence comparisons suggest two genes, one represented by three, M58336, M64337, M72894, and the other by one mRNA, AY049920. Among the first group, M72894 and M64337 show a single 3rd position difference among 830 bases of coding region and only 1 difference in 210 bases of the 3' UTR, while M58336 and M64337 show a single 2nd position difference among 566 bases that occurs at a 5 consecutive same-base segment, a common sequencing error. Conversely, alignment of the sequence representing the second gene, AY049920 with a member of the first group, M64337 exhibits the characteristic difference distribution indicating distinct genes, 6 in the 1st, 6 in the 2nd, and 19 in the 3rd position (p < 0.005). A review of the literature cited for these sequences [23,24] does not suggest any conclusions regarding the relationship of these genes, therefore the alignments stand as the only criterion to judge the accuracy of the clustering.

Cluster 4c of WS100 and WS200 represent two distinct genes of the ferritin iron storage protein. UniGene correctly clustered three of the sequences together while keeping separate a singleton that represents the second gene. See Figure 1.

### Cluster 4e

Cluster 4e contains mRNA sequences encoding phosphoenolpyruvate carboxylase. Sequence analysis indicates two distinct genes, one represented by three, D13998, AB008541, AB008542, and the other by one mRNA, AB008540. Pairwise comparisons among the members of the first group indicate very few differences, whereas the alignment of the unique mRNA, AB008540 with one member of the first group, D13998, exhibited 9 differences in the 1st, 6 in the 2nd, and 42 in the 3rd position over 2900 bases of coding region (p < 0.005). The literature cited for these sequences concurs with this relationship among these genes [25,26].

Cluster 4e of WS100 and UniGene represents two distinct genes of phosphoenolpyruvate carboxylase. Neither of the three methods correctly separated the unique sequence and WS200, likely as a result of short sequences, kept two as singletons that should belong to the cluster. See Figure 1.

### Cluster 4f

Cluster 4f contains mRNA sequences encoding omega-3-fatty acid desaturase. Alignments indicate two distinct genes each represented by two mRNA sequences. The first, AB105887, AY204710 show 0 differences in the 1st position, 2 in the 2nd, and 4 in the 3rd (p > 0.05, 0.21). The second, AB105886, AY204711 show 1 difference in the 1st, 0 in the 2nd, and 2 in the 3rd position (p > 0.05, 0.57). Conversely, the comparison between groups, AY204711 and AY204710 for example, exhibited differences as 8 in the 1st position, 6 in the 2nd, and 32 in the 3rd (p < 0.005). All of these sequences are unpublished direct submissions.

Cluster 4f of WS100 and UniGene represents two distinct genes of omega-3-fatty acid desaturase. Only WS200 correctly distinguished between the sequences representing separate genes. See Figure 1.

### Cluster 4g

Cluster 4g contains mRNA sequences encoding phosphoenolpyruvate carboxylase kinase. Sequence comparisons suggest two distinct genes each represented by two mRNA sequences. The first, AY144182, AY143660 show only 1 difference over 825 bases of coding region. The second, AY144184, AY373033 are 100% identical over 825 bases of coding region. However, the comparison between groups, AY144182 and AY373033 for example, show differences characteristic of divergent genes, 12 in the 1st position, 5 in the 2nd, and 36 in the 3rd (p < 0.005). This conclusion is supported in the cited literature [27,28].

Cluster 4g of WS100 represents two distinct genes of phosphoenolpyruvate carboxylase kinase. Both WS200 and UniGene correctly distinguished between the sequences representing separate genes. See Figure 1.

### Cluster 4h

Cluster 4h contains mRNA sequences encoding urease. Alignments suggest two distinct genes each represented by two mRNA sequences. The first, AY230156, AJ276866 show only 1 difference over 2514 bases of coding region. The second, AY230157, S69179 are show only 2 differences over 379 bases of coding region. However, the comparison between groups, AY230157 and AJ276866 for example, show differences characteristic of divergent genes, 46 in the 1st position, 31 in the 2nd, and 100 in the 3rd over 1740 bases of coding region (p < 0.005). This conclusion is supported in the cited literature [29,30].

Cluster 4h of WS100 represents two distinct genes encoding urease, one the ubiquitous urease and the other embryo-specific. Only UniGene correctly distinguished between the sequences representing these separate genes. WS200 was further off the mark by including one of the embryo-specific sequences in the ubiquitous cluster and the other as a singleton. See Figure 4.

### Cluster 4i

Cluster 4i contains mRNA sequences encoding glycinin and conglycinin storage protein subunits. As previously stated, sequence similarities that are shared between the subunit classes make the type of sequence comparison performed here inconclusive. However, it is clear from a review of the literature cited for these sequences [6,31] that two, X05652 and X02626 represent glycinin subunit and the other two, J01296 and J01295 represent conglycinin subunits.

Cluster 4i of WS100 represents two separate types of seed storage proteins glycinin and conglycinin. This is not surprising since these proteins share some domain similarities. It has been suggested that this reflects requirements for construction, stability, or utilization of these proteins [6]. WS200 comes closest to being correct by separating as singletons the two sequences representing conglycinin genes from a cluster containing the two glycinin mRNA sequences. See Figure 5.

### Type I and Type II Errors

UniGene was correct in 6 of 12 clusters, WS100 was correct in 2 of 12, and WS200 in 4 of 12. (Table 2). Among the 12 clusters that exhibited differences (where a reasonable estimate could be made regarding the validity of the cluster either from sequence analysis or the literature) 6 were determined to be correct clusters by either WS100 or WS200. Four of those were also correctly clustered by UniGene. Only 2 of the 12 was determined to be correctly assigned by UniGene that was not correctly assigned by either WS100 or WS200 and 4 others were determined to be incorrectly clustered by all three methods. This suggests that, although PECT may not be significantly more accurate than UniGene, combining results from different stringencies of clustering can provide the individual researcher making use of clusters the option of determining which stringency provides the best result for the gene or gene family of interest.

## Conclusion

Neither of the clustering methods described here can be shown to provide results that clearly indicate that it is performing the desired task of separating gene sequences into clusters representing unique genes more accurately the other. However, our results on clustering known sequences (mRNAs) provide evidence that using different stringencies or a hierarchical method of clustering should produce results that are reliable for more genes. UniGene clustered 6 of 12 correctly and PECT clustered 6 of 12 correctly when results for both stringencies are combined. In addition, such a hierarchical method will provide individual researchers more information about sequence relationships within gene families, superfamilies or even between functionally distinct genes. Patterns revealed by such analysis should provide significant insight into genome evolution. In addition, the advantage of a fast clustering algorithm such as PECT is the ability to repeat the clustering runs with different criteria, or to cluster only related libraries grouped together in different ways. UniGene takes months, is unchangeable except for updates, and as shown in this study, is no more reliable than a fast clustering algorithm. The next step is to apply PECT to the entire EST database for soybean and assign these mRNA clusters to the appropriate EST cluster to determine the validity of clustering sequences that are shorter on average and have greater error frequency. Additionally, automation of the analysis of clusters, specifically pairwise alignments and mismatch coding location, will allow the generation of valid non-redundant gene sets for any species represented in the database.

## Methods

### Generating Clusters

Accession numbers of the 837 soybean mRNA sequences from a previous UniGene *Glycine max *build were extracted and used as the starting dataset. The dataset was used twice to generate gene-specific clusters at two different sequence matching stringencies, 100% match over window size 100 (WS100) and 100% match over window size 200 (WS200). WS100 represents the lower stringency criterion and WS200 the higher.

### Analysis of Clusters

All non-singleton clusters generated from both stringencies were compared to one another as well as to UniGene. The largest clusters (greater than 3 members) that exhibit differences between the three methods were analyzed by pairwise alignment of sequences with accompanying amino acid sequence using bl2seq [32]. Each mismatch between mRNA sequences that occurred in the coding region was scored for its codon position. The assumption was made that differences as a result of error should be distributed evenly among the first second and third positions, whereas differences as a result of divergence (indicating a paralogous relationship) should be more frequent in the third degenerate position of the codon. X^2 ^was used to provide a probability that the differences represented a uniform distribution among the codon positions. The references cited for the mRNA sequences were reviewed to determine the conclusions of the submitting authors, when available. Together these two analyses were used to make a judgement as to the correctness of each mRNA sequence in its cluster.

### Assignment of Type I and Type II Error

For each cluster examined the type of error made by each stringency and UniGene was recorded. Type I and Type II errors are revised from the convention of Burke et al.[33] to be consistent with statistical hypothesis testing. Briefly, the null hypothesis is that any two mRNA sequences represent distinct genes and belong in separate clusters. A type I error (rejecting a true null) would put two mRNA sequences in the same cluster when they actually represent different genes (overrclustering). A type II error (failing to reject a false null) would keep two mRNA sequences in separate clusters when they actually represent the same gene (underclustering).

## Authors' contributions

RLF participated in the conception and design of the study, carried out the sequence alignment analysis and literature review, performed the statistical analysis, and drafted the manuscript. FE participated in the conception, design, and development of the algorithm PECT and coordinated the generation of cluster data. All authors read and approved the final manuscript.

## Figures and Tables

**Table 1:** Cluster Size Profiles. The number of clusters of each size are given for all three methods.

**Cluster size**	**WS100**	**WS200**	**UniGene**
1	519	602	581
2	76	60	61
3	20	15	18
4	7	3	5
5	4	4	4
6	2	0	1
7	0	2	0
8	1	1	1
9	1	0	1
12	1	0	0
16	0	1	0
17	1	0	1
**Total**	632	688	673
**Total >1 (NS)**	113	86	92

**Table 2:** Type I and Type II Errors. Type I and Type II errors are tallied for each of clusters size 4 or greater that exhibited differences among the three methods.

Clusters			Error Type		
WS100	WS200		WS100	WS200	UniGene

12	7a 5d	**Figure 2**	type I type II	type I type II	4 type I type II

**9**	7b s s		3 type I	type II	3 type I

6b	**3a** **3b**		type I	none	none

6a	**3i** **3j**		type I	none	none

5f	**4a** **s**		none	type II	type II

3o 3aa	3o 2ggg+s	**Figure 3**	type I	type I type II	type I

4i	2hhh s s	**Figure 4**	type I	type I	type I type II

4h	3m s	**Figure 5**	type I	type I type II	none

4g	**2v** **2w**		type I	none	none

**4f**	2yy 2zz		type I	none	type I

**4e**	2xx s s		none	type I 2 type II	none

4c	4c	**3s+s**	type I	type I	none
